# Trends in reported malaria cases and the effects of malaria control in the Democratic Republic of the Congo

**DOI:** 10.1371/journal.pone.0219853

**Published:** 2019-07-25

**Authors:** Filippo Lechthaler, Barbara Matthys, Giulia Lechthaler-Felber, Joris Losimba Likwela, Hypolite Muhindo Mavoko, Junior Matangila Rika, Meschac Mutombo Mutombo, Laura Ruckstuhl, Joanna Barczyk, Estifanos Shargie, Helen Prytherch, Christian Lengeler

**Affiliations:** 1 Swiss Centre for International Health, Swiss Tropical and Public Health Institute, Basel, Canton of Basel Stadt, Switzerland; 2 University of Basel, Basel, Canton of Basel Stadt, Switzerland; 3 School of Agricultural, Forest and Food Sciences, Bern University of Applied Sciences, Zollikofen, Canton of Bern, Switzerland; 4 Faculty of Business and Economics, University of Basel, Basel, Canton of Basel Stadt, Switzerland; 5 Soins de Santé en Milieu Rural (non-profit organization SANRU), Kinshasa, Democratic Republic of the Congo; 6 Tropical Medicine Department, University of Kinshasa, Kinshasa, Democratic Republic of the Congo; 7 National Malaria Control Program, Ministry of Health, Kinshasa, Democratic Republic of the Congo; 8 Epidemiology and Public Health Department, Swiss Tropical and Public Health Institute, Basel, Canton of Basel Stadt, Switzerland; 9 The Global Fund to fight AIDS, Tuberculosis, and Malaria, Geneva, Canton of Geneva, Switzerland; Instituto Rene Rachou, BRAZIL

## Abstract

**Background:**

Considerable upscaling of malaria control efforts have taken place over the last 15 years in the Democratic Republic of Congo, the country with the second highest malaria case load after Nigeria. Malaria control interventions have been strengthened in line with the Millenium Development Goals. We analysed the effects of these interventions on malaria cases at health facility level, using a retrospective trend analysis of malaria cases between 2005 and 2014. Data were collected from outpatient and laboratory registers based on a sample of 175 health facilities that represents all eco-epidemiological malaria settings across the country.

**Methods:**

We applied a time series analysis to assess trends of suspected and confirmed malaria cases, by health province and for different age groups. A linear panel regression model controlled for non-malaria outpatient cases, rain fall, nightlight intensity, health province and time fixed effects, was used to examine the relationship between the interventions and malaria case occurrences, as well as test positivity rates.

**Results:**

Overall, recorded suspected and confirmed malaria cases in the DRC have increased. The sharp increase in confirmed cases from 2010 coincides with the introduction of the new treatment policy and the resulting scale-up of diagnostic testing. Controlling for confounding factors, the introduction of rapid diagnostic tests (RDTs) was significantly associated with the number of tested and confirmed cases. The test positivity rate fluctuated around 40% without showing any trend.

**Conclusion:**

The sharp increase in confirmed malaria cases from 2010 is unlikely to be due to a resurgence of malaria, but is clearly associated with improved diagnostic availability, mainly the introduction of RDTs. Before that, a great part of malaria cases were treated based on clinical suspicion. This finding points to a better detection of cases that potentially contributed to improved case management. Furthermore, the expansion of diagnostic testing along with the increase in confirmed cases implies that before 2010, cases were underreported, and that the accuracy of routine data to describe malaria incidence has improved.

## Introduction

The United Nations Millennium Development Goals (MDGs) and the Roll Back Malaria Partnership’s call to “halt by 2015 and begin to reverse the incidence of malaria” [1, 2] catalysed an international funding commitment to malaria control in sub-Saharan Africa from 2000 onwards. As a result, prevalence of *Plasmodium falciparum* in Africa has declined from 40% in 1900–1929 to 24% in 2010–2015 [3]. Infection prevalence and case incidence was significantly reduced in 32 highly endemic countries in Africa, with increased coverage of insecticide-treated nets (ITNs) as the main contributor [4].

Key control interventions, notably ITNs, indoor residual spraying (IRS), rapid diagnostic tests (RDTs) combined with prompt treatment of confirmed malaria cases with artemisinin-based combination therapies (ACTs), and intermittent preventive treatment in pregnancy (IPTp) were massively scaled up during the last 15 years. In total, US$ 960 million in 2005 and US$ 2.5 billion in 2014 were globally invested in malaria programmes, focusing largely on commodities (ITNs, IRS, RDTs and ACTs). Malaria diagnostic testing increased from 36% of suspected cases reported by countries in 2005 to 41% in 2010 and 65% in 2014 in the WHO African Region [5].

Considerable upscaling of control efforts has also taken place in the Democratic Republic of the Congo (DRC), the country with the second highest malaria case load in the world after Nigeria. As a result of these massive efforts, modelling outcomes predict a clear reduction in the number of cases and deaths from malaria [6].

Malaria control in the DRC focused in the early 20^th^ century on breeding sites management, individual protection with mosquito nets, screening of windows, and prophylaxis with quinine. An exhaustive review of malaria control developments has been prepared in 2014 [7]. The first vector control measures in Kinshasa in 1976 were initiated as an agreement between the United States Agency for International Development (USAID) and the Government of the country called Zaïre at the time. The National Malaria Control Program (NMCP) was then launched nationally in 1998. From 2004 onwards, the main contributor of a substantially increased funding for malaria in the DRC was The Global Fund to Fight AIDS, Tuberculosis and Malaria (Global Fund), with contributions from the President’s Malaria Initiative (PMI), the United Kingdom’s Department for International Development (DFID) and the United Nations International Children’s Emergency Fund (UNICEF).

The distribution of ITNs started in antenatal care facilities within the Expanded Program on Immunization (EPI) in 2003. A policy on free ITNs for pregnant women and children aged below 5 years was adopted in 2006 and expanded through mass distributions of ITNs within the universal coverage campaign starting in 2008. To-date, over 64 million ITNs have been distributed in all provinces in the country. IPTp with Sulfadoxine-Pyrimethamine (SP) was adopted in 2003 and introduced in 2005. The Ministry of Health adopted a free treatment policy of ACTs for all in 2006 and introduced Artesunate/amodiaquine (ASAQ) as first-line treatment and Artemether-Lumefantine (AL) as second-line treatment in 2010. All 516 health zones were supposed to be covered by ACTs in 2012, according to the policy. Case management with ACTs by community health workers was implemented in 2008, and parasitological diagnosis using RDTs was introduced at health facility and community levels in 2010. In parallel, a network of 11 sentinel sites for an integrated surveillance of priority diseases, including malaria, was established in 2003, with an expansion to the 26 new provinces in 2016. Entomological surveillance of insecticide resistance has been conducted in the selected sentinel sites of Bas-Congo, Kinshasa, Equateur and South Kivu since 2009.

In 2015, the NMCP and its partners, with support from the Global Fund evaluated the current malaria situation in the country to draw conclusions on whether progress had been achieved. The execution of this large-scale assessment was contracted through the faith-based Nongovernmental Organization “Soins de Santé Primaire en Milieu Rural” (SANRU) to the Division of Health Management Information System of the Ministry of Health, the Kinshasa School of Public Health and the Swiss Tropical and Public Health Institute (Swiss TPH), with substantial inputs from the NMCP. The assessment followed a malaria “rapid impact assessment (RIA)” approach–an approach developed by the Wold Health Organization and used to assess malaria intervention impact in a number of settings [8–10]. We report here the results of this rapid impact assessment to collect and evaluate data on malaria from outpatient and laboratory registers in the DRC between 2005 and 2014.

## Materials and methods

This assessment is entirely based on primary data from outpatient and laboratory registers at health service delivery points. We conducted an in-depth analysis of the effects of malaria control interventions on malaria-related indicators at health facility level by (i) assessing the trends in suspected, tested, and confirmed malaria cases as well as non-malaria cases between 2005 and 2014 and (ii) investigating the relationship between malaria control interventions and the occurrence of suspected, tested and confirmed malaria cases, with a focus on parasitological diagnosis.

### The setting

The DRC is the largest country in Africa South of the Sahara, with a total land mass of over 2.3 million km^2^. With an estimated 85 million inhabitants [11], it is also one of the most populated countries. This enormous landmass combined with poor infrastructure means that obtaining reliable health information is challenging. In recent years some improvements on the routine health data monitoring system has meant that routine data have become more available and more reliable. But with remaining data quality issues, it was decided for the present study to do a primary data collection based on patient and laboratory registers in health facilities, rather than to rely on existing Health Management Information System (HMIS) data. The malaria morbidity assessment was carried out jointly with another assessment of mortality of HIV/Aids, tuberculosis and malaria at hospital level.

### Data collection—Malaria cases

A suspected malaria case was defined as any case being classified as malaria in the clinical diagnosis in the outpatient register, irrespective of having finally received a parasitological test or not. A tested case was defined as a suspected malaria case that received a parasitological test. A confirmed malaria case was defined as one where malaria was parasitologically demonstrated, either by microscopy or by RDT. Malaria case in the text refers to confirmed malaria case. Testing coverage was defined as the number of tested cases divided by the number of suspected cases. Malaria test positivity rate was defined as the number of positive tests (RDTs or microscopy) divided by the number of malaria tests (RDT or microscopy) performed. The non-malaria cases were obtained by subtracting suspected cases from all-cause outpatient cases.

Data was obtained from laboratory and outpatient register abstraction based on a nation-wide health facility survey that was conducted together with the NMCP and the Division of Health Management Information System of the Ministry of Health from December 2015 to March 2016. In a country the size of the DRC with limited geographic accessibility of health zones, a random sampling was impossible to carry out. Hence, health facilities were selected based on a purposive sampling strategy which was guided by the following criteria: (1) representation of the three eco-epidemiological malaria settings (tropical, equatorial and mountain zone), (2) availability of parasitological diagnostic tools, (3) level of service use and reporting completeness (between 2010 and 2014), and (4) general accessibility.

**Fig 1:**
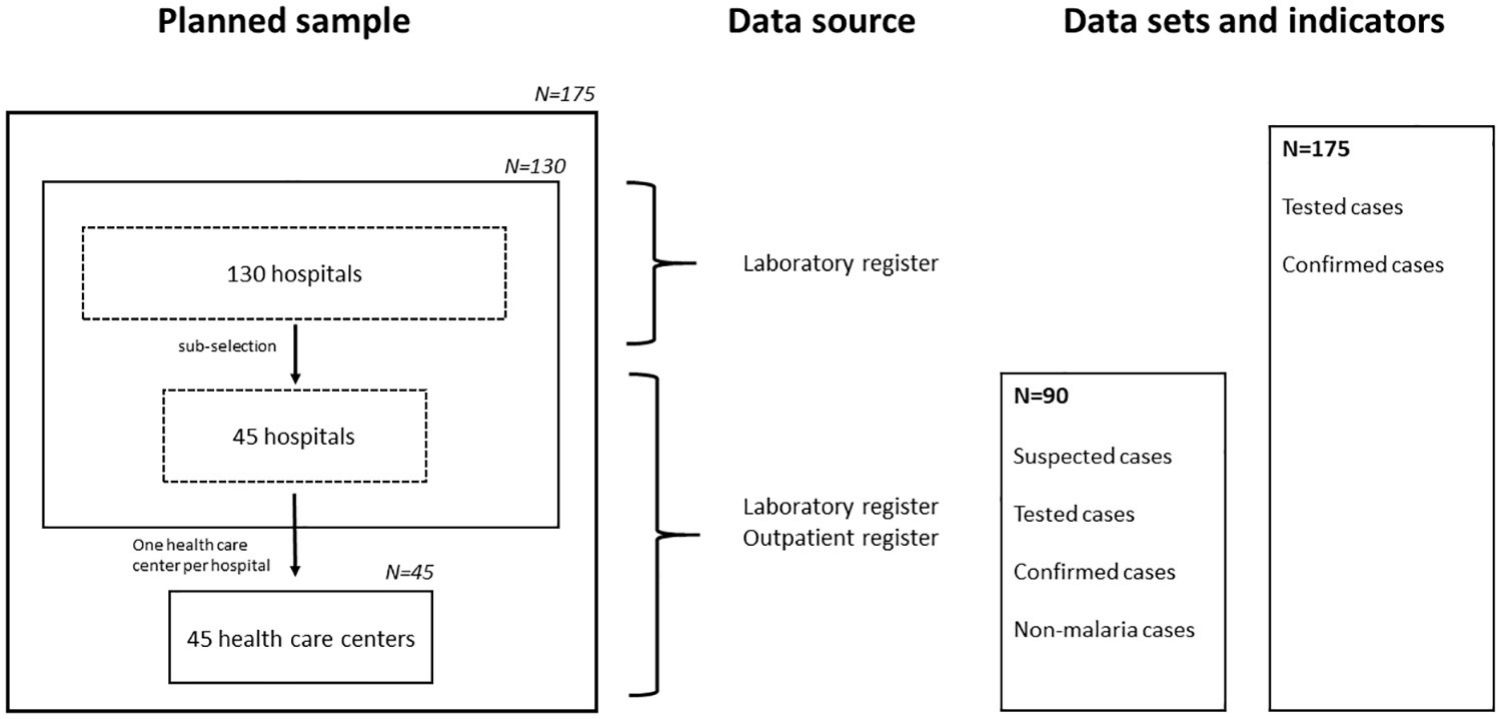
Planned sampling strategy, data sources, data sets and indicators.

The sampling strategy is visualized in Fig 1. In a first step, 130 hospitals were selected for laboratory register abstraction in line with the concomitant study on hospital mortality covering all 26 Provincial Health Directorates of the DRC: 47 hospitals in the equatorial zone, 60 hospitals in the tropical zones and 23 hospitals in mountain zone. Laboratory registers were available in tabular forms with most documenting the total number of tests performed and test results per type of test (microscopy or RDT) per day. To refine the information obtained from laboratory registers, a subset of 45 hospitals (15 per epidemiological setting) was purposively selected from the 130 initial hospitals to collect case-specific information from outpatient registers, which allowed differentiating between suspected, tested and confirmed malaria cases as well as non-malaria cases. For each of the 45 hospitals in the subsample, one appertaining health care center in the same health zone was randomly selected for outpatient and laboratory register abstraction. Outpatient registers were available in tabular form as printed or hand-written documents with one line being typically assigned per one patient visit.

Data was considered as adequate and included in the analysis if information on the indicators selected for this study was available for more than 60% of the months in the study period, and if less than 30% of consecutive months were missing. For each facility monthly totals of outpatient visits and laboratory tests were calculated. The number of outpatient cases (suspected and confirmed malaria cases as well as non-malaria related cases) was stratified by 4 age groups: 0 to 4 years, 5 to 9 years, 10 to 14 years, and more than 14 years. Laboratory records represent total monthly aggregates as they could not be broken down by age.

### Data on intervention coverage

Data on ITN distributions, either through routine ANC and EPI distributions, or mass campaigns, were obtained from the NMCP records. Data on the number of distributed ITN were available from 2006 and covered all 26 health provinces. The availability of RDTs in the visited health facilities was assessed by reviewing stock records.

### Analytical approach and statistical methods

The attribution of changes in malaria cases to a particular intervention based on routine surveillance data is challenging due to complex causal pathways and the many co-interventions occurring during the period under review. Nevertheless, tt has been shown that for the evaluation of public health (and specifically malaria-related) interventions based on HMIS data, quasi-experimental designs based on a plausibility approach are appropriate designs to assess programme performance and impact [12, 13]. Other than in randomized control trials, in plausibility assessments the choice of a control group is guided by opportunistic criteria, taking advantage of the existing data structure [14]. For the present study, we made use of the relatively uniform nation-wide expansion of diagnostic testing through the change in the national policy in 2010 that made parasitological confirmation mandatory, by creating historical control groups represented by pre-intervention subsamples. This set-up allowed comparing outcomes before and after the intervention at national level. Furthermore, applying a multivariate regression approach and controlling for confounding factors, we exploited the staggered implementation pattern for RDTs and ITNs at sub-national level, giving rise to internal controls represented by areas where the malaria control intervention had started later in time. The methodological details and statistical methods are described in the following paragraphs.

In a first step, data were analysed based on a historical comparison using the following approaches:

(1) *Descriptive analysis of trends*: simple descriptive interpretation of trends in suspected, tested, and confirmed malaria cases at the national level were applied using plotted trends. Short-term fluctuations were removed to better interpret long-run trends using the Hodrick Prescott filter with monthly smoothing parameter [15].
(2) *Pre-post intervention comparison*: average suspected, tested, and confirmed malaria cases were estimated before and after the main intervention (introduction of RDTs) and tested statistically for evidence in changes over time using Welch’s t-tests. Case numbers were assessed at national level as well as by Health Province.
  The main limitation of approaches (1) and (2) is the lack of a counterfactual that describes and compares changes in testing and clinical outcomes over the evaluation period in the absence of the intervention. This requires the strong assumption that the pre-intervention period is similar to the post-intervention period in all relevant characteristics (except for the exposure to the intervention itself) implying that all changes in malaria cases over time are attributable to the introduction of RDTs. To refine the description of malaria changes and its attribution to the interventions, the following methods were additionally applied:
(3) *Interrupted time series*: A segmented regression of interrupted time series was used to compare observed to predicted values after the intervention (introduction of RDTs in 2010) at national level, versus before 2010, assuming a continuation of the pre-intervention time trend [16, 17]. More specifically, the time series of interest was used to estimate a regression model with an underlying trend, which is interrupted by the introduction of RDTs. This model was then applied to predict a hypothetical scenario (or counterfactual) for the post-intervention period, under which the intervention had not taken place and the trend continued unchanged (i.e. in the absence of the intervention, given the pre-intervention trend). The comparison was based on the predicted values in the total number of cases (all case consults, suspected, tested and confirmed) in 2014 as well as on the mean number of annual cases during the post-intervention period (2010–2014). An Autoregressive Integrated Moving Average (ARIMA) model was used as the time series regression. Model selection was guided by the Akaike Information Criterion (AIC) value [18]. The segmented model controls for possible time trends of the indicator during the full period, an additive change in the indicator through the intervention, and a time trend in the indicator after the intervention.
*(4) Subnational analysis*: Testing outcomes were analysed at sub-national level to explore the relationship between interventions and malaria testing outcomes, which allowed exploiting variations in the onset of the interventions that began at different times in different health provinces. Subnational-disaggregation further enabled confounding to be dealt with through the inclusion of probable contributing factors in a multivariate regression. A panel linear regression was used to investigate the association between tested malaria cases, confirmed malaria cases and the test positivity rate, and antimalarial interventions (RDT availability and ITNs distributed through mass campaign) based on province data at yearly frequency. The regression models were defined as follows:
  $$y_{ijt}={trend}_{t}+\gamma_{i}+{trend}_{t}\times \theta_{j}+X_{ijt}\delta +\varepsilon_{ijt},$$
  where *y*_*ijt*_ represents the log of tested malaria cases, the log of confirmed malaria cases or the positive test (slide or microscopy) rate respectively for Health Province *i* in political province *j* in year *t*. *trend*_*t*_ is a time trend, *γ*_*i*_ denotes fixed effect coefficient of Health Province *i*, and the interaction term *trend*_*t*_ × *θ*_*j*_ represents province-specific time trends. Eleven Health Provinces represent the administrative division of the country before 2016 and are used to control for regional trends. 26 Health Provinces represent the unit of analysis and correspond with the current administrative division of the DRC. *X*_*ijt*_ is a vector of country-year covariates controlling for RDT availability, a dummy variable indicating years of ITN mass campaigns, rainfall, economic progress and the log of non-malaria related cases. Non-malaria related outpatient cases from register data were included to control for trends in general health service use. Satellite data on night-time lights distributed by the National Oceanic and Atmospheric Administration (NOAA) were used as a proxy for economic progress [19, 20]. Light intensity reflected by indoor and outdoor activities is closely connected to productive human activity and economic progress [21, 22] which in turn is expected to favour malaria control efforts. Annual grid-level satellite data on nightlight intensity is available on a 30 arc-second grid cell resolution, which is equivalent to approximately 0.86 square kilometres at the equator, and reported as a six-bit digital number (an integer between 0 = no light and 63) for every grid. Data disturbed by moonlight data, auroral activity, forest fires, clouds and other factors were removed. Information on precipitation was obtained from the CRU TS3.10 dataset for monthly climatological variables with a resolution of a 300 arc-minutes [23]. Boundary coordinates for each Health Province in the DRC allowed localizing them on the geo-referenced satellite data (for night light and precipitation) and identifying raster grids which fully or partly fall within those Health Provinces boundary boxes. By averaging the respective values of grid cells inside a given Health Province, a measure for rain and night lights was obtained for each Health Province. Values were aggregated at the yearly level for statistical analysis.

Robustness checks were performed using two additional specifications: first, we included the log of the number of ITNs distributed during routine and mass campaign in year *t* in Health Province *i* and second, we included a one-year lag of ITN mass campaign distribution in order to account for possible delays in the impact.

R version 3.4.2 and STATA 15 were used for data analysis.

### Ethical clearance

Ethical approval was obtained by the ethical committee of the School of Public Health at the University of Kinshasa (Approval Nr: ESP/CE/105/2015). Specific measures for treatment of ill individuals were not required as only retrospective data was used from health facility registers.

## Results

### Realized sample and implications for data analysis

Of the 175 sites where laboratory data was collected (full sample), 131 met the inclusion criteria and were considered for analysis. From the sub-selection of 90 sites in which data from the outpatient registers were recorded, 84 were included for analysis. In 59 sites from 21 Health Provinces, both laboratory data and outpatient data could be considered. However, information on parasitological tests could be linked individually to the outpatient cases for only 32% of the retained outpatient sites (27 sites).

To maximize the geographic coverage of the included sites as well as optimally exploit the available information from the collected data, different sub-samples have been used for different analytical purposes:

1. *At national level*: first, tested and confirmed cases from laboratory registers were assessed based on the realized full sample (N = 131). Second, the realized sub-selection (N = 84) was used to additionally analyse trends in suspected, and non-malaria cases that were abstracted from outpatient registers. Last, the analysis of tested and confirmed cases per all cause consults, as well as suspected, tested and confirmed cases per age-group was realized for the 27 sites were outpatient data could be individually linked to test results (N = 27).
2. *At sub-national level*: analysis at health province levels including the panel regression was based on the sub-sample that combines laboratory and outpatient data (N = 59) which enabled joint analysis and interpretation of indicators from laboratory (tested and confirmed cases) and outpatient registers (suspected and non-malaria cases). Due to the low number of sites (N = 27), sub-national analysis could not take individual patient characteristics into account.

### Interventions

A total of 60.5 million ITNs were distributed through the NMCP between 2006 and 2014. The majority of those ITNs (51.2 million) were distributed through mass campaigns targeting the entire population in areas with stable malaria endemicity. In addition, 9.3 million ITNs were distributed through a routine distribution strategy targeting antenatal and preschool consultations.

The percentage of sampled facilities with RDTs available for at least one quarter per year increased from almost zero to 8% in 2009, with a sharp increase to 56% in 2010, reaching 100% by the end of 2014.

### National trends of malaria cases

**Fig 2:**
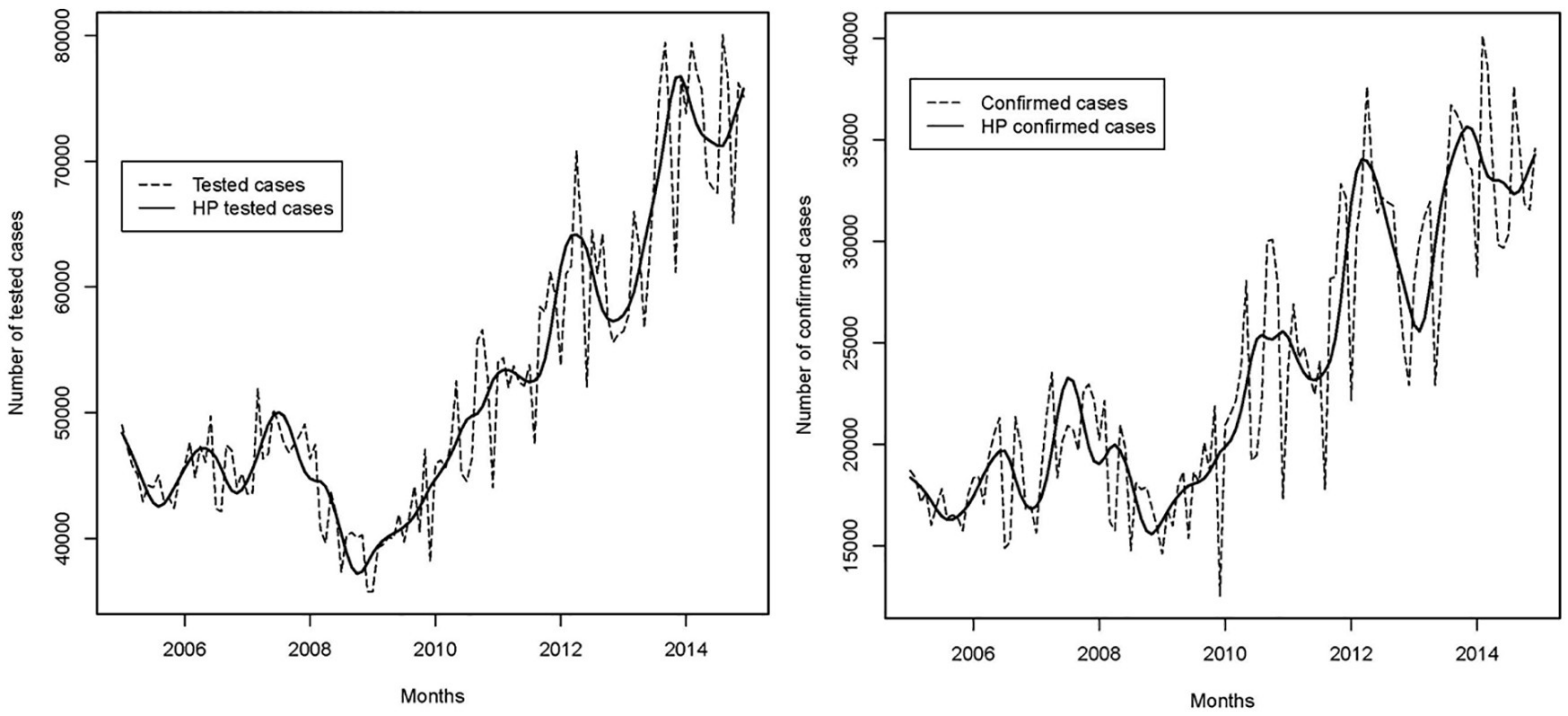
Figs 2a and 2b. Trends of a) tested, and b) confirmed malaria cases (RDT and microscopy), Democratic Republic of the Congo, 2005–2014. Source: laboratory register, full sample (N = 131). HP signifies Hodrick-Prescott filter.

Applying the Hodrick-Prescott filter to remove short-term fluctuations in nationally aggregated data, Fig 2 shows that generally the number of monthly tested and confirmed malaria cases abstracted from laboratory registers increased between 2005 and 2014, with some degree of inter-annual variation. An accelerate growth rate for both tested and confirmed cases, can be observed between the years 2008 and 2010. Apart from inter-annual variations, suspected cases and non-malaria related cases have followed a rather constant positive trend as compared to confirmed cases recorded from laboratory registers (Fig 2a and 2b).

**Fig 3:**
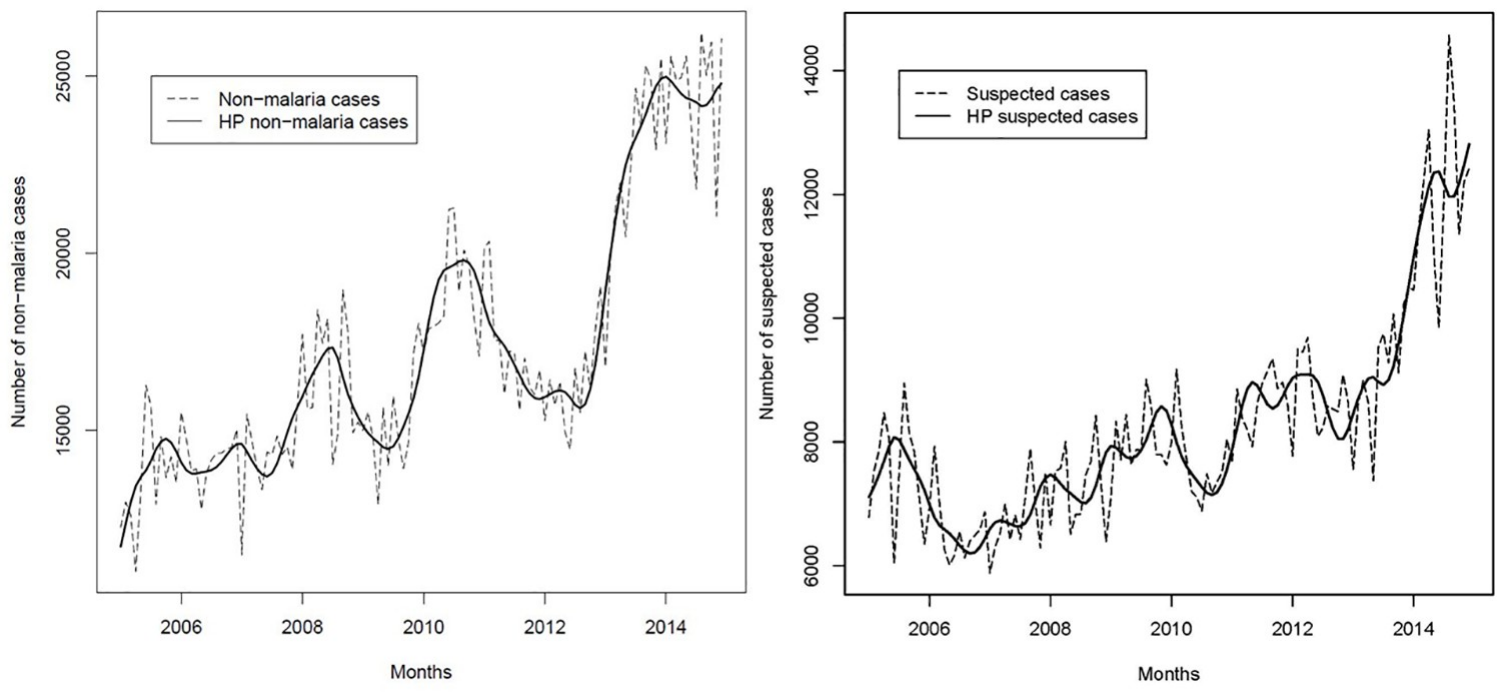
Figs 3a and 3b. Trends of a) non-malaria and b) suspected cases, Democratic Republic of the Congo, 2005–2014. Source: outpatient register, sub-sample (N = 84).

The numbers of monthly suspected malaria cases and non-malaria related cases recorded from outpatient registers increased between 2005 and 2014 (Fig 3a and 3b).

**Fig 4:**
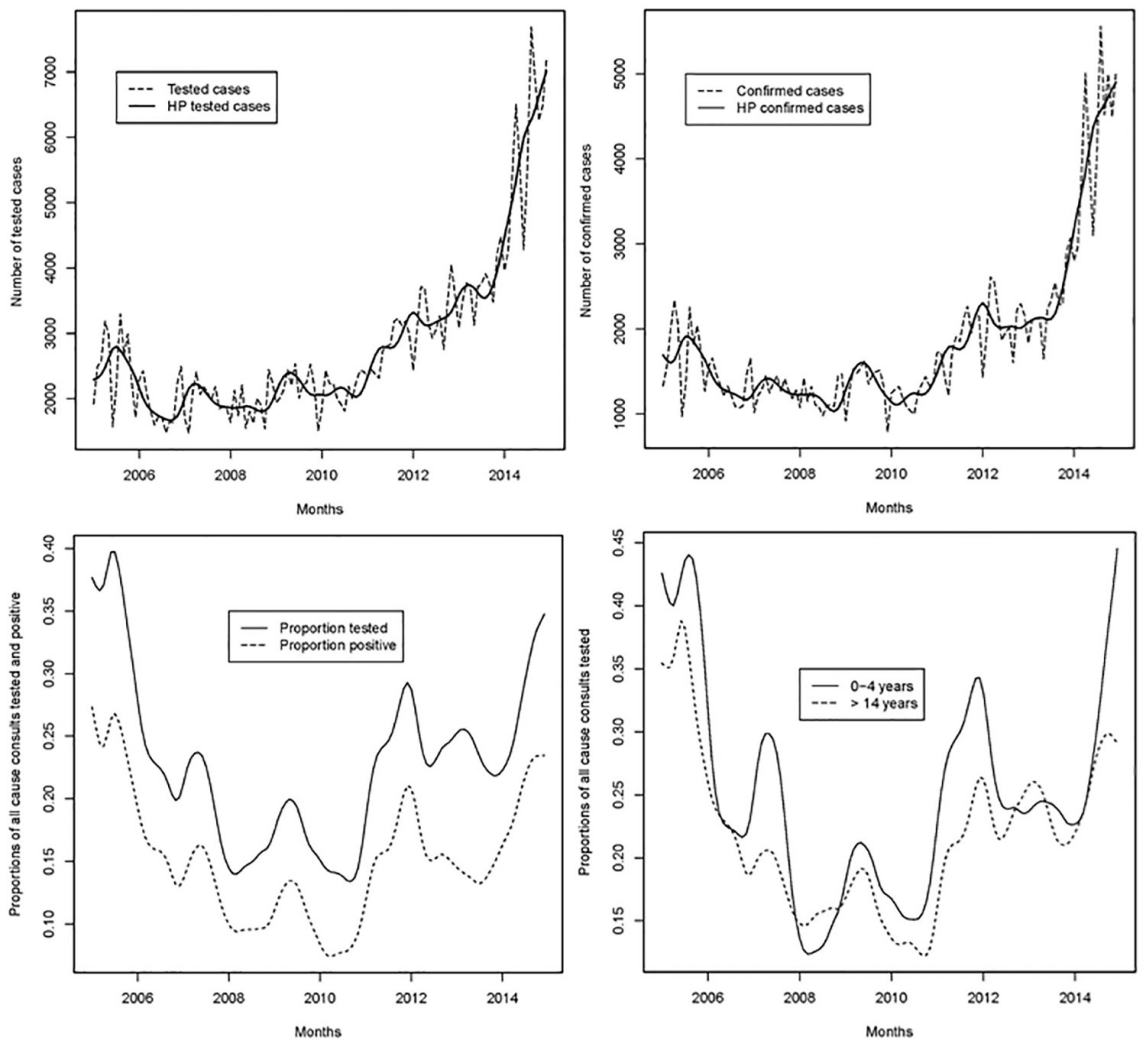
Figs 4a-4d. Trends in a) tested and b) confirmed cases from RDT and microscopy. Proportions of c) all consults tested and positive (RDT and microscopy) and d) proportions of all consults tested, stratified by age group, Democratic Republic of the Congo, 2005–2014. Source: outpatient register, sub-sample of sites where parasitological tests could be linked individually to the outpatient register (N = 27).

Fig 4 shows malaria-related indicators calculated from the sub-sample of sites where parasitological tests could be linked individually to the outpatient register. Tested and confirmed cases in Fig 4a and 4b affirm the insights from the laboratory register: while numbers were generally increasing between the years 2005 and 2010, the corresponding growth rates hiked between the year 2008 and 2010. Looking at the proportions of all cause consults that were tested (RDT and microscopy) in Fig 4c, it can be observed that this share was relatively high in the year 2005 (between 0.35 and 0.4) and decreased in the subsequent years. The proportion reached a low point between the 2008 and 2010 (between 0.15 and 0.2) and caught up between the years 2010 and 2014 returning to its initial value. The proportion of all cause consults with positive test results (RDT and microscopy) revealed a similar pattern (Fig 4c). Fig 4d presents the proportions of all cause consults tested (RDT and microscopy) for two age groups (0–4 years and > 4 years) indicating that the proportion was slightly higher for smaller children (0–4 years) most of the time.

**Fig 5:**
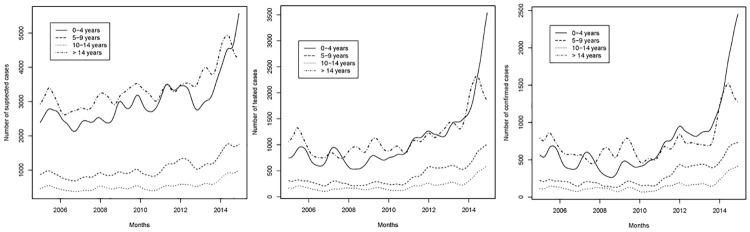
Figs 5a-5c. Trend of a) suspected, b) tested, and c) confirmed malaria cases (RDT and microscopy), by age group, Democratic Republic of the Congo 2005–2014. Source: outpatient register, sub-sample of sites where parasitological tests could be linked individually to the outpatient register (N = 27).

Fig 5a–5c present monthly suspected, tested (RDT and microscopy) and confirmed malaria cases from the outpatient register, disaggregated by age. It can be observed that young children (0–4 years) and adults (>14 years) accounted for the major part of suspected, tested and confirmed cases. The sharp increase in confirmed cases after 2010 is visible for all age groups but was particularly pronounced for children younger than 5 years.

**Fig 6:**
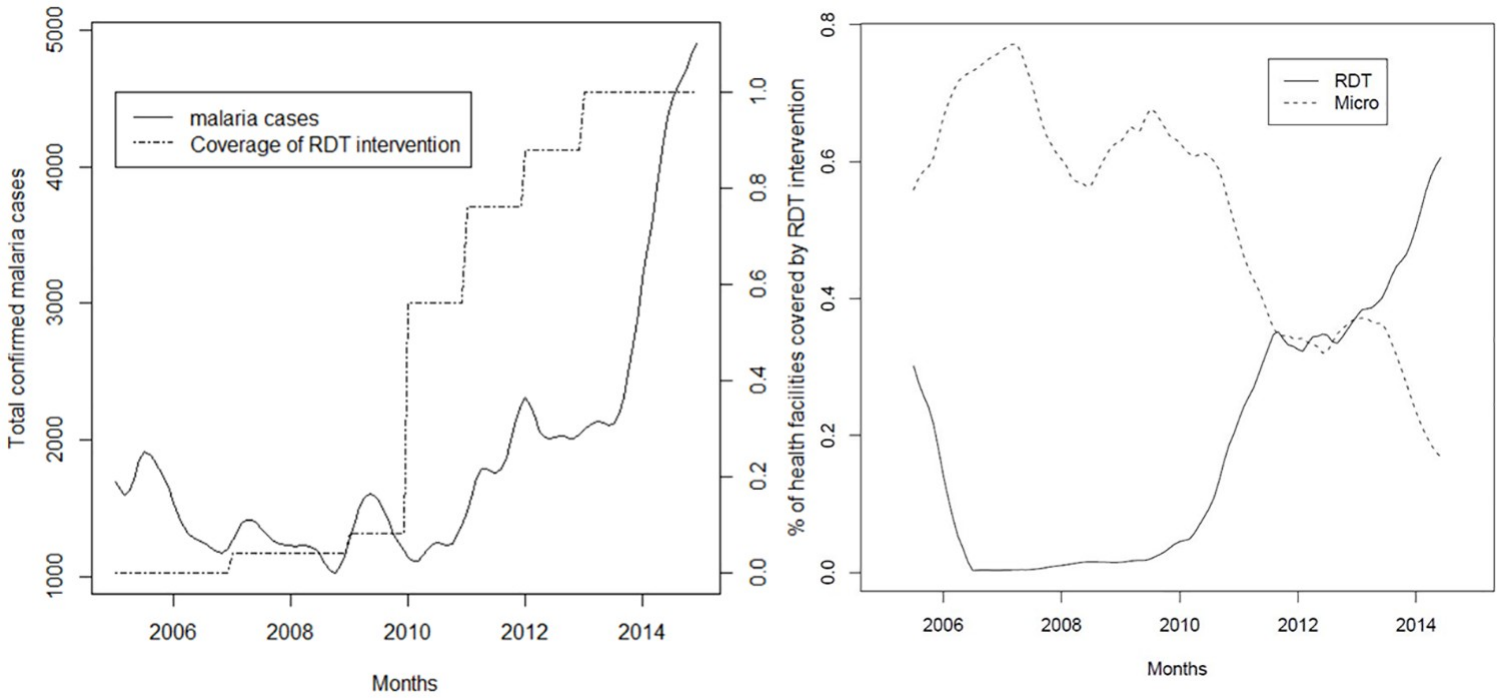
Fig 6a. Confirmed malaria cases (RDT and microscopy) against the percentage of health facilities with RDTs available in the Democratic Republic of Congo between 2005 and 2014. Fig 6b. Proportion of suspected malaria cases receiving a parasitological test (RDT or microscopy), differentiated by RDT and microscopy in the Democratic Republic of Congo between 2005 and 2014. Source: outpatient register, sub-sample of sites where parasitological tests could be linked individually to the outpatient register (N = 27).

Fig 6a shows confirmed malaria cases (RDT and microscopy) against the percentage of health facilities with RDTs available. The increase in RDT coverage is linked to the increase in confirmed malaria cases, with a sharp increase in 2010 coinciding with the scale-up of RDTs and the introduction of the national policy making parasitological confirmation mandatory.

Fig 6b presents the proportion of suspected cases receiving a parasitological test either by RDTs or microscopy, calculated from the outpatient register and differentiated by RDT and microscopy. Testing coverage was generally high on average (fluctuating between 60% and 80%) with RDTs continuously replacing microscopy starting from 2010.

**Fig 7:**
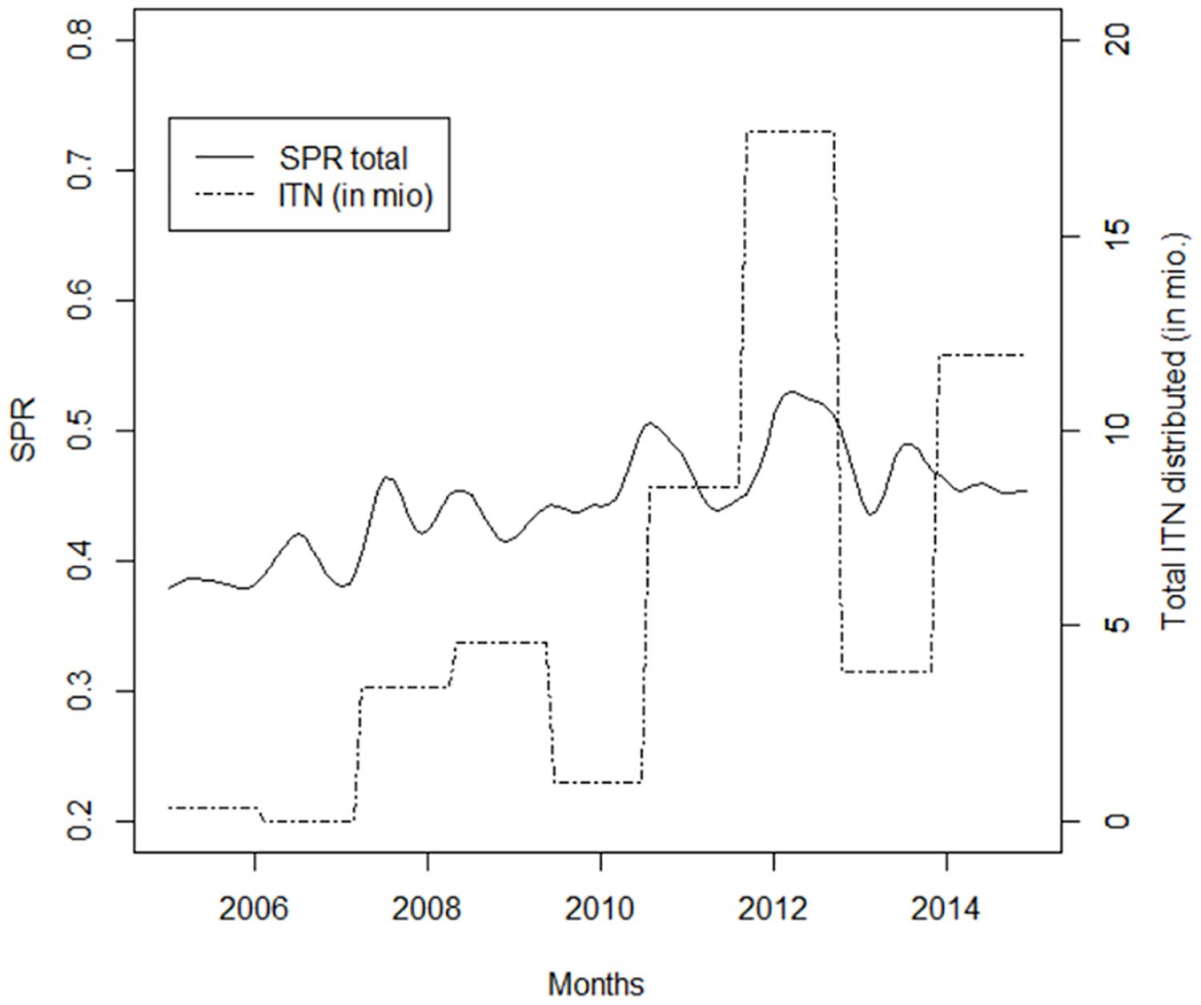
Test positivity rate (RDTs and microscopy) against the number of ITNs distributed in the Democratic Republic of Congo between 2005 and 2014. Source: laboratory register, full sample (N = 131).

Fig 7 plots the test positivity rate of RDTs and microscopy against the number of ITNs distributed. Nationally aggregated data from the NMCP on distributed ITNs indicate that in 2011 and 2012 a total number of 29 million nets were distributed. The test positivity rate fluctuating around 40% did not show any considerable trend.

Nationally aggregated cases (all case consults, suspected cases and confirmed cases from outpatient registers, and tested and confirmed cases from laboratory registers) were evaluated against the introduction of the national policy on RDTs in 2010, comparing average monthly cases before and after this transition point. It was of course expected that through the expansion of malaria diagnostic testing the number of confirmed cases would also increase. Table 1 shows that for the sub-samples that were based on the outpatient register, all indicators (all cases, non-malaria related cases, suspected and confirmed malaria cases) have increased significantly between the pre- and post-intervention period, throughout all age groups (columns 1, 2, and 3). Looking at all ages, confirmed cases increased by 51%, suspected cases by 26%, and non-malaria cases by 32%. The increase in confirmed cases was particularly strong for children below 5 years, with an increase of 82% in average monthly cases. Older age groups exhibited lower case increase rates post-intervention: confirmed cases increased by 65% in the second age group (5–9 years), by 48% for the third age group (10–14 years) and 23% for the fourth age group (>14 years).

**Table 1:** Percentage change in malaria and non-malaria related indicators comparing pre- and post-intervention periods as well as predicted and observed values, Democratic Republic of the Congo, 2005–2014. Source: tested and confirmed cases from laboratory register, full sample (N = 131). All cases and suspected cases from outpatient register, sub-sample (N = 84). Confirmed cases from outpatient register, sub-sample of sites where parasitological tests could be linked individually to the outpatient register (N = 27).

			Monthly means and changes			Change in predicted vs observed	
Register	Age group	Indicator	Preintervention mean (2005–2010)	Postintervention mean (2011–2014)	% changes	Mean 2010–2014	2014
			(1)	(2)	(3)	(4)	(5)
Outpatient	<5 years	All cases, <5	6.751	9,524	41%*	27%*	56%
		Suspected cases <5	2.591	3,428	32%*	12%*	41%
		Confirmed cases, <5	446	813	82%*	338%*	1,804%
	5–9 years	Suspected cases, 5–9	860	1,218	42%*	17%	52%
		Confirmed cases, 5–9	182	300	65%*	76%	235%
	10–14 years	Suspected cases, 10–14	460	603	31%*	29%	90%
		Confirmed cases, 10–14	107	158	48%*	61%	225%
	>14 years	Suspected cases, >14	3,046	3,725	22%*	7%	29%
		Confirmed cases, >14	617	760	23%*	19%	91%
	All ages	All cases, all ages	22,007	29,126	32%*	17%	41%
		All non-malaria cases, all ages	14,736	19,931	35%*	21%	41%
		Suspected cases, all ages	7,271	9,196	26%*	13%	42%
		Confirmed cases, all ages	1,358	2,046	51%*	76%	240%
Laboratory	All ages	Tested cases, microscopy	31,582	35,366	12%*	-8%	-27%
		Tested cases, RDT	1,010	9,223	813%*	2,676&	4,884%
		Tested, cases, microscopy & RDT	44,080	60,626	38%*	52%	91%
		Confirmed cases, microscopy	14,082	17,872	27%*	-15%	-43%
		Confirmed cases, RDT	142	3,953	2,681%*	Inf%	Inf%
		Confirmed cases, blood film & RDT	18,430	28,747	56*	37%	52%

Note:

* p < 0.05.

Significance in column (3) is based on a t-test, for the pre-intervention period and the post-intervention period. A significance implies that the pre-intervention and post-intervention monthly mean average are significantly different. Inf. Stands for infinity (estimates did not converge)

Indicators calculated from the sub-sample based on laboratory registers (tested and confirmed cases) revealed that the increase in tested and confirmed cases was particularly driven by the use of RDTs, with a percentage change of 813% and 2681% respectively as compared to only 12% and 27% for microscopy. The part of the test results that could not be assigned to a specific diagnostic method increased by 38% and 56% respectively. The number of tested and confirmed cases calculated from laboratory registers also indicates that the testing coverage for microscopy increased as well but to a lesser extent than for RDTs (12% for tested cased and 38% for confirmed cases).

Table 1 presents the results from the segmented regression: the comparison of predicted and observed values were based on the total number of malaria cases in 2014 (column 4) as well as on the mean number of annual cases during the full post-intervention period (2010–2014, column 5). Overall, predicted counterfactual values, as calculated from the segmented regression, in suspected and confirmed as well as non-malaria related cases were lower in 2014 as well as during 2010–2014 than the observed values. This spread was particularly pronounced for the confirmed malaria cases. In fact, for the period between 2010 and 2014 (column 4), the observed number of confirmed malaria cases for all ages was 76% higher than the predicted number, whereas the observed suspected malaria and non-malaria related cases exceed their prediction by 13% to 21%. This effect is even more pronounced when looking only at the year 2014 (column 5). The strong increase in confirmed malaria cases seems to be driven by an increase in confirmed cases among children below 5 years of age, with the observed value being 338% higher than the prediction for the period 2010–2014. Following the pattern in the pre- and post-intervention comparison, this effect decreased with an increase in the age group reaching 19% for the population >14 years. Looking at the indicators calculated from the laboratory register (tested and confirmed cases), the model predicted a strong acceleration of RDT applications after 2010 (by almost 5000% for the number of tested cases in the year 2014) whereas tests based on microscopy were expected to decrease (by -27% for tested cases and -43% of confirmed cases by the year 2014).

### Disaggregated analysis

Following the analysis of national level data, Table 2 presents pre- and post-intervention monthly means of non-malaria cases, laboratory tested and laboratory confirmed malaria cases based on the sub-sample that combines laboratory and outpatient registers for 59 sites in 21 Health Provinces. Results generally confirmed the findings from the national level analysis. In 17 out of 21 Health Provinces, confirmed malaria cases significantly increased after 2010. In 13 out of these 17 Health Provinces the increase in confirmed malaria cases was higher than for the non-malaria cases.

**Table 2:** Comparing pre- and post-intervention monthly mean number of non-malaria cases, laboratory tested, and laboratory confirmed malaria cases (RDT and microscopy) per Health Province, in the Democratic Republic of the Congo between 2005 and 2014. Source: sub sample combining laboratory (tested and confirmed cases) and outpatient registers (non-malaria cases) (N = 59).

Province	Provincial Health Directorate	Year of RDT introduction	Non-malaria cases			Laboratory tested cases			Laboratory confirmed cases		
			Pre-introd.	Post-introd.	%-change	Pre-introd.	Post-introd.	%-change	Pre-introd.	Post-introd.	%-change
			(1)	(2)	(3)	(4)	(5)	(6)	(7)	(8)	(9)
Bandundu	Kwango	2010	528	710	34%*	1,069	1,642	54%*	567	899	59%*
	Kwilu	2011	1,404	1,235	-12%*	3,280	2,869	-13%	981	1,535	57%*
	Mai Ndombe	2010	115	194	68%*	916	699	-24%*	224	422	89%*
Bas-Congo	Kongo Central	2012	1,928	2,157	12%*	2,748	2,949	7%	1,057	1,301	23%*
Equateur	Equateur	2010	452	311	-31%*	2,279	4,977	118%*	2,208	4,608	109%*
	Mongala	2013	523	317	-39%*	612	403	-34%*	526	269	-49%*
	Nord Ubangi	2010	328	274	-17%*	375	1,632	341%*	219	952	334%*
	Tshuapa	2013	316	275	-13%*	453	488	8%	193	250	30%*
Kasaï Occidental	Kasaï	2013	599	463	-23%	2,056	1,987	-3%*	824	1,370	66%*
	Kasaï Central	2010	402	491	22%*	1,059	1,276	20%*	555	758	37%*
Kasaï Oriental	Lomami	2010	197	335	70%*	1,484	2,043	38%*	609	930	53%*
Katanga	Haut Katanga	2010	561	337	-40%*	2,935	3,352	14%*	1,312	1,506	15%
	Haut Lomami	2011	141	142	0%*	1,055	295	-72%*	129	136	5%
	Lualabal	2007	154	102	-34%*	1,145	831	-27%*	923	602	-35%*
	Tanganyika	2011	698	939	35%*	226	2,013	791%*	137	894	552%
Kinshasa City	Kinshasa	2011	606	813	34%	7,901	11,526	46%*	3,581	4,298	20%*
Kivu	Maniema	2010	520	678	30%*	1,077	1,085	1%	280	354	27%*
	Nord Kivu	2010	3,815	7,097	86%*	5,352	11,079	107%*	1,508	3,885	158%*
	Sud Kivu	2011	2,998	2,523	-16%*	4,425	4,503	2%	1,196	1,028	-14%*
Orientale	Haut Uelé	2010	765	977	28%*	641	789	23%*	403	510	26%*
	Ituri	2010	261	436	67%*	353	818	132%*	126	396	214%*
	Tshopo	2010	178	206	16%	953	1,921	201%*	215	689	221%*

Note:

* P < 0.05.

Significance column (3) is based on a t-test for the pre-intervention period and the post-intervention period. A significance implies that the pre-intervention and post-intervention monthly mean averages are significantly different.

Table 3 presents multivariate regression results for tested and confirmed malaria cases, as well as the test positivity rates based on the same sub-sample. The introduction of RDTs was unsurprisingly significantly associated with the number of laboratory tested cases, as well as the confirmed cases. In fact, controlling for confounding factors, the introduction of the RDTs is linked to an average increase of 37% in tested cases and around 25% in confirmed cases. The number of non-malaria cases was positively associated with the laboratory tested and confirmed cases but only significant for the laboratory tested cases. Nightlight intensity was negatively and precipitation was positively related to the number of tested and confirmed cases. However, these results were not significant at the 10% level. Only the test positivity rate was significantly related to precipitation. The introduction of ITN was not significantly associated with either of the dependent variables.

**Table 3:** Results from a multivariate regression model for laboratory tested and confirmed malaria cases and test positivity rates (RDT and microscopy), in the Democratic Republic of Congo. Source: sub sample combining laboratory (tested and confirmed cases) and outpatient registers (non-malaria cases) (N = 59).

	Laboratory tested		Laboratory confirmed		Test positivity rate	
	(1)	(2)	(3)	(4)	(5)	(6)
RDT introduction	0.370** (0.159)	0.369** (0.157)	0.248** (0.110)	0.247** (0.109)	-0.054 (0.040)	-0.054 (0.041)
Ln (non malaria cases)	0.140* (0.081)	0.131 (0.088)	0.111 (0.068)	0.099 (0.074)	-0.017 (0.030)	-0.020 (0.031)
Ln (night lights)	-0.159 (0.205)	-0.145 (0.210)	-0.115 (0.164)	-0.096 (0.163)	-0.029 (0.095)	-0.025 (0.096)
Ln (precipitation)	0.105 (0.349)	0.105 (0.351)	0.429 (0.382)	0.429 (0.382)	0.188* (0.100)	0.188* (0.100)
LLIN introduction		0.067 (0.077)		0.088 (0.080)		0.020 (0.019)
Year FE	Yes	Yes	Yes	Yes	Yes	Yes
Provincial Trend	Yes	Yes	Yes	Yes	Yes	Yes
Obs.	193	193	193	193	193	193
R^2^	0.323	0.325	0.368	0.373	0.276	0.278

Note:

* p < 0.1,

** p < 0.05,

*** p < 0.01.

Robust standard errors in parantheses (). RMSE is the root mean square error. RDT intro is a dummy variable equal one for the year equal or after the RDT introduction. LLIN intro is a dummy variable equal one for the years where a LLIN distribution mass campaign occurred and for the 2 following years. Ln() = logarithmic function. FE = fixed effect.

## Discussion

This study is a first attempt to use routine data to empirically assess malaria control interventions in the DRC covering the whole country with a focus on parasitological diagnosis. The study assessed possible links between trends in outpatient malaria cases at health care facilities and key malaria control interventions in the DRC from 2005 to 2014. It made use of outpatient and laboratory records that were obtained through a large-scale retrospective survey in 175 health facilities distributed over the entire country. Associations between parasitological diagnosis in the context of RDT scale up and malaria morbidity reports have been rarely investigated so far, probably because RDT scale up rather refers to a commodity, not considering the behaviour of the health care provider. Secondly, intervention scale up itself affects the numerator and denominator (positive/tested) dramatically and in such a way that is difficult to draw conclusions on the burden of malaria. The majority of research has focused rather on effects of commodity-based interventions such as ITNs and diagnosis and treatment.

The most striking finding was the increasing number of monthly confirmed malaria cases, with a strong rise from 2010 onwards, whereby the trend of suspected cases and non-malaria related cases showed a rather linear increase. Also, the test positivity rate remained rather constant at 40%, indicating sustained high transmission. Assumptions on possible underlying factors for this increase of confirmed cases and of confounders are discussed below.

Our findings demonstrate that the increase of confirmed malaria cases is most probably a result of the strengthening of specific components of the health system over the past years, in particular, reinforcing the diagnostic and case management capacity, coupled with improved recording and reporting practices, and probably increases in patient consultations. Indeed, results show that tested cases (microscopy and RDT) as well as proportions of all case consults that were tested have increased considerably after 2010. The proportions of tested cases were already high in 2005, which reflects the general finding that tested and confirmed cases were higher in 2005 than during the period between 2006 and 2010. A possible reason for this is a data availability bias: registers were generally more likely to be missing during the early years of the observation period. Hence, they may have been only available in a health facility where data management practices and general performance during these early years was of superior quality (see limitation section below). Results derived from outpatient registers also indicate that after the scale-up of RDTs, microscopy was increasingly replaced by RDTs. This finding is in contrast with the crude comparison of indicators from laboratory registers before and after 2010 which indicate that tested and confirmed cases rather remained at a certain level after the scale-up of RDTs. A possible reason is that the laboratory registers typically documented counts of tests performed per day, thereby including cases that were repeatedly tested using both an RDT and microscopy. As a consequence these cases were double counted, also described from other studies in the DRC [24]. The application of the segmented regression to tested and confirmed cases from laboratory registers, however, revealed that indeed microscopy was expected to decrease after 2010.

Prior to the introduction of RDTs and the new malaria treatment guidelines around 2010, the number of confirmed cases consisted of microscopy-based biological confirmation, and of a fraction of clinically diagnosed cases that were incorrectly recorded as parasitological confirmed cases. The application of RDTs involves shorter test procedures, allowing more tests to be performed within a given time period. Moreover, RDTs are adapted to remote and resource-constrained health areas with weak or absent laboratory infrastructure, and often a lack of supervision. Thus, compared to the pre-intervention period, the introduction of RDTs enabled health providers to expand the number of biological tests and confirmations, which accelerated the increase in confirmed cases. The findings from this study are consistent with an analysis of reported malaria incidence rates between 2010 and 2014 based on national HMIS data in the DRC that showed an increase of the number of reported confirmed cases, with a stable test positivity rate [24]. The potential reporting bias in our study was excluded by collecting data recorded from primary sources (registers). The increase of confirmed cases thus implied that cases were considerably underreported before 2010, and that the number of correctly diagnosed cases has augmented.

Mandatory parasitological confirmation, along with the increased coverage of parasitological tests, was significantly associated with the number of tested and confirmed cases. When controlling for confounding factors, scale-up of RDTs was significantly associated with an average yearly increase of 37% of tested cases and of 25% of confirmed cases. Comparing monthly averages of confirmed cases before and after January 2010, an increase of 65% was observed for children aged 5–9 years and of 48% for children aged 10–14 years. This fraction might be even higher since RDTs were found to miss 13% of malaria infections among children aged below five years compared to polymerase chain reaction (PCR) method in the DRC [25]. A possible reason for higher confirmed case number for children below five years is targeting of RDTs at this age group, even though patients of all ages should receive a parasitological test since 2007 [26]. Indeed, proportion of all cause consults that have been tested by microscopy or RDT was slightly higher for children below five years as compared to the other age groups which may explain a part of the age difference in confirmed malaria cases.

Our interpretation is in line with recent similar trends: the World Malaria Report 2018 stated a massive scale-up of the use of RDTs in Africa, coupled with efforts to strengthen the HMIS. National routine data have improved in terms of quantity and quality, and reporting rates to national surveillance systems have increased. WHO estimates for confirmed malaria cases from the public health sector alone are still lower than the real number of confirmed cases, and are thus underestimated for various African countries [27]. There is thus reason to assume that the actual number of reported cases in the DRC has not yet reached the actual number of cases.

An increase of malaria cases from hospital-based pediatric admission data was also observed in Malawi between 2000 and 2010 [28], Uganda between 1999 and 2009 [29], and Kenya between 2003 and 2009 [30], and a substantial rise of confirmed malaria cases between 2005 and 2010 in Togo. The authors of the Togo study presume underreporting of cases prior to the introduction of the new case management policy. Better access to malaria interventions (subsidized treatment, extension to community-based health service provision), strengthened capacity of health professionals and of the reporting system were moreover cited to contribute [31]. The strengthening of the national surveillance system was reported as contributor for the increase of tested malaria cases in Lusaka, Zambia. The introduction of the open software “District Health Management Information System” (DHIS2) allowed visualising reported trends and achievements of milestones in malaria case management, which encouraged the health staff in their efforts [32].

The majority of evaluations could however show that a scale up of preventive interventions such as ITNs resulted in a decrease of confirmed cases. Interventions to increase the coverage of ITNs, IRS and ACTs supported by PMI in 15 sub-Saharan African countries were associated with a 12% reduction in all-cause child mortality [33]. Achievements on reducing the malaria morbidity and mortality through key malaria control interventions at health facility and community level were seen in Senegal [34], Rwanda [35], Zambia [36], Ethiopia [9], Ghana [8], and in Zimbabwe [37]. Nevertheless, confirmed malaria cases will continue to rise with the increased use of RDTs to test cases of febrile illness among children aged below five years.

A visual interpretation of the trend of the test positivity rate and the numbers of ITNs distributed indicates that the level of the test positivity rate decreased the first year after mass distribution campaigns and then increased in the subsequent year. The regression results for the test positivity rate (as well as for tested and confirmed cases) were however not significantly associated with the introduction of ITNs. Similar trends were observed in a community-based cross-sectional survey in the former Health Province of Kasaï Occidental, where a high malaria prevalence was found among children aged under 5 years of age, despite a high ITN coverage after a mass distribution campaign [38]. ITN use may not be optimal for example due to a lack of appropriate place to hang the net, reluctance to use it, displacement due to insecurity, use for other purposes or simply that insecticide resistance has rendered conventional ITNs less effective ([39], [40]).

This study had clear limitations. Objections to use routine surveillance data to investigate malaria program impact are well established, including a large potential for measurement errors and confounding [12]. Reviewing information from primary data sources (registers) at health facility level allowed at least overcoming sources of reporting bias. However, part of the data was missing or could not be linked or cross-checked between different sources (e.g. outpatient with laboratory registers), and the analyses were applied to different subsets of the pre-selected facilities. As a consequence a part of the analysis was based on reduced sub-samples–for example the age stratification–and were therefore not representative for the full sample. The potential bias due to data exclusion is probably moderate as the fraction of excluded sites was limited, apart for the subsample where parasitological tests could be linked individually to the outpatient cases. Furthermore, all estimation related to the use of RDTs before 2010 are subject to high uncertainty as only a very small number of sites were using this type of diagnostic test. Main reasons for incomplete data were missing registers due to poor archiving practices and external events such as political unrest and natural disasters. Earlier records were more likely to be missing than recent ones.

The sampling strategy applied strict eligibility criteria and targeted facilities with minimum performance standard (with particular emphasis on reporting completeness between 2010 and 2014 and delivery of parasitological diagnosis with microscopy and/or RDTs). There is a possibility that trends would be different if conclusion criteria were minimised a more representative sample of facilities would have been analysed. However, the consistency of our findings with an analysis of national HMIS trends gives some reassurance that our findings are representative of the national picture. A bigger likely problem is confounding, for example variations in the baseline transmission, changes in service use, and reporting practices. Attention has been paid to control for confounding factors based on an interrupted time series approach and multivariate regressions. By controlling for period- and province- specific trends, regional fixed effects, as well as for a number of probable confounding variables, this analysis has tried at least to minimize the potential for confounding.

## Conclusion

This study analysed routine surveillance data from outpatient registers in the DRC between 2005 and 2014, and investigated potential effects of key antimalarial interventions. A positive trend of recorded outpatient cases between 2005 and 2014 was observed, with a pronounced acceleration of confirmed cases around 2010. This trend strongly coincides with the introduction of the new treatment policy and the resulting scale-up of diagnostic testing with RDTs. The relative increase of confirmed cases is particularly marked among children below five years of age. Results suggest that the fraction of correctly diagnosed cases has augmented, and that recording (and reporting) practices have improved.

With a typically poor service contact of the population, available surveillance data capture only a part of the actual malaria transmission in the DRC. The introduction of DHIS2 in the DRC opens new opportunities to better evaluate potential effects of interventions on key indicators. An improved recording of cases and systematic link to laboratory data at individual level would substantially contribute towards this.
